# Association of Milk Consumption and Vitamin D Status in the US Population by Ethnicity: NHANES 2001–2010 Analysis

**DOI:** 10.3390/nu12123720

**Published:** 2020-12-02

**Authors:** Moises Torres-Gonzalez, Christopher J. Cifelli, Sanjiv Agarwal, Victor L Fulgoni

**Affiliations:** 1 National Dairy Council, Rosemont, IL 60018, USA; Chris.Cifelli@dairy.org; 2 NutriScience LLC, East Norriton, PA 19403, USA; agarwal47@yahoo.com; 3 Nutrition Impact LLC, Battle Creek, MI 49014, USA; VIC3RD@aol.com

**Keywords:** 25-hydroxyvitamin D (25(OH)D), Mexican–American, Other Hispanic, non-Hispanic White, non-Hispanic Black

## Abstract

Vitamin D has been identified as a nutrient of public health concern, and higher intake of natural or fortified food sources of vitamin D, such as milk, are encouraged by the 2015–2020 Dietary Guidelines for Americans. We, therefore, examined the association of milk consumption and vitamin D status in the United States (US) population. Twenty-four-hour dietary recall data and serum 25(OH)D concentrations were obtained from the National Health and Nutrition Examination Survey 2001–2010 and were analyzed by linear and logistic regression after adjusting for anthropometric and demographic variables. Significance was set at *p* < 0.05. Approximately 57–80% children and 42–60% adults were milk consumers. Milk intake (especially reduced-fat, low fat and no-fat milk) was positively associated (*p*_linear trend_ < 0.05) with serum vitamin D status and with a 31–42% higher probability of meeting recommended serum vitamin D (>50 nmol/L) levels among all age groups. Serum vitamin D status was also associated with both type and amount of milk intake depending upon the age and ethnicity. In conclusion, the results indicate that milk consumers consistently have higher serum vitamin D levels and higher probability of meeting recommended levels. Therefore, increasing milk intake may be an effective strategy to improve the vitamin D status of the US population.

## 1. Introduction

Vitamin D (calciferol) is a fat-soluble vitamin, photosynthesized in the skin by the action of solar ultraviolet (UV) B radiation. It is naturally found in only a few foods, such as fish-liver oils, fatty fishes, mushrooms, egg yolks, and liver [1]. Vitamin D is known to regulate calcium and phosphorus absorption and, therefore, it has been traditionally associated with skeletal health, and its deficiency increases the risk of rickets in children, and osteoporosis, fractures and falls in adults [1,2,3,4,5]. Emerging evidence suggests that vitamin D deficiency may also be linked to the development of cardiovascular disease, hypertension, diabetes, metabolic syndrome, cancer, multiple sclerosis, rheumatoid arthritis, and sarcopenia [6,7,8,9,10,11,12,13].

The United States (US) Centers for Disease Control and Prevention (CDC) Second National Report on Biochemical Indicators of Diet and Nutrition in the US population reported that, in 2003–2006, approximately 8% of the population aged 1 year and older were at risk for vitamin D deficiency (VDD), which varied by age, gender, or race/ethnicity, and was as high as 31% in non-Hispanic blacks [14]. More recent analysis of NHANES 2011–2014 data, which oversampled Asian, non-Hispanic black, and Hispanic individuals to obtain reliable estimates for these population subgroups, indicated that 18.3% Americans aged 1+ years were at risk of vitamin D inadequacy (VDI) based on serum levels [15]. While there were no significant gender differences, there was a quadratic trend for risk of VDI by age and was higher for adults 20–39 years than for children 1–5 years and for seniors ≥ 60 years [15]. VDI also varied by ethnicity and was lowest among non-Hispanic White, followed by Hispanics and Asians, and was highest among non-Hispanic Blacks [15]. Vitamin D levels in humans are assessed by the determining serum 25-hydroxyvitamin D (25(OH)D) concentrations. Serum 25(OH)D levels of less than 30 nmol/L (12 ng/mL) are considered at risk for deficiency, serum levels between 30 and less than 50 nmol/L (12 to less than 20 ng/mL) are considered at risk for inadequacy; serum levels between 50–75 nmol/L (20–30 ng/mL) are considered sufficient; serum levels greater than 125 nmol/L (50 ng/mL) may be of potential concern [1].

Although, vitamin D is produced endogenously by exposure to sunlight, seasonal variations, cultural practices, and physiologic factors can impair sunlight-induced synthesis of vitamin D. The current usual dietary intakes of vitamin D among US adults aged 19+ years are 5.3 μg/d for males and 4.1 μg/d for females, and 92% males and over 97% females are below the Estimated Average Requirement (EAR) [16]. Similarly, vitamin D intakes of 90 to 93% in male and 95 to over 97% in female children aged 4–18 years are also below the EAR [16]. Accordingly, the 2015–2020 Dietary Guidelines for Americans (DGA) identified vitamin D as a “nutrient of public health concern” as it is under-consumed to an extent that may lead to adverse health outcomes [17]. The Scientific Report of the 2020 Dietary Guidelines Advisory Committee reaffirmed vitamin D is under-consumed and is of public health concern [18]. The EAR of vitamin D is 400 IU (10 μg) for ages 1+ years and the Recommended Dietary Allowance (RDA) is 600 IU (15 μg) for ages 1–70 years and 800 IU (20 μg) for ages 70+ years [1]. Vitamin D can be acquired from fortified foods and dietary supplements [1,17]. In the American diet, fortified foods are a main source of the vitamin D [1,19,20]. Varieties of foods fortified with vitamin D in the US include dairy products (mostly milk), cereals and fruit juices. Milk is voluntarily fortified with 400 IU per quart (or 385 IU/L) of vitamin D [19] and almost all fluid milks are fortified with vitamin D in the US market [21]. Indeed, DGA encourages a higher intake of food sources of vitamin D, such as milk, to meet the requirements [17].

The objective of the present investigation was to determine the association of milk consumption and vitamin D status in the US population, and to examine if milk consumers have better vitamin D status as compared to non-consumers. We hypothesized that higher milk consumption is associated with better vitamin D status and that milk consumers, regardless of the type of milk consumed, have better vitamin D status compared to non-milk consumers across all age groups.

## 2. Materials and Methods

### 2.1. Database & Subjects

Data from five separate cycles of What We Eat In America (WWEIA), the dietary intake component of the National Health and Nutrition Examination Survey (NHANES), a continuous survey conducted by the National Center for Health Statistics (NCHS), were used (2001–2010). NHANES data are collected using a complex stratified multistage cluster sampling probability design. A detailed description of the subject recruitment, survey design, and data collection procedures are available online [22] and all data obtained for this study are publicly available at: http://www.cdc.gov/nchs/nhanes/. Dietary intake data with reliable 24-h recall dietary interviews (day 1 data only) from 33,672 participants (17,132 male and 16,540 female; 4061 aged 2–8 years, 8700 aged 9–18 years, 17,457 aged 19–70 years and 3454 aged 71–99 years; 7827 Mexican American, 2094 Other Hispanic, 14,525 non-Hispanic White, 7739 non-Hispanic Black, 1487 of other ethnicity) were used after with exclusions for unreliable data (*n* = 5690), aged < 2 years (*n* = 3079), pregnant or lactating females (*n* = 1272), missing serum vitamin D data (*n* = 5733), missing Poverty Index Ratio (PIR; *n* = 2808), missing Body Mass Index (BMI; *n* = 836) or zero calorie intake (*n* = 2). All participants or proxies (i.e., parents or guardians) provided written informed consent and the Research Ethics Review Board at the NCHS approved the survey protocol. This study was a secondary data analysis which lacked personal identifiers and, therefore, did not require Institutional Review Board review.

### 2.2. Estimation of Dietary Intake

Intake of milk was assessed using the sum of Food Patterns Equivalents Database (FPED) variable “D_Milk” as cup equivalents/day from associated WWEIA categories:

- Whole Milk—1002 (Milk, Whole), 1202 (Flavored milk, whole)
- Reduced-fat Milk—1004 (Milk, reduced-fat), 1204 (Flavored milk, reduced-fat)
- Low-fat Milk—1006 (Milk, low-fat), 1206 (Flavored milk, low-fat)
- Non-fat Milk—1008 (Milk, nonfat), 1208 (Flavored milk, non-fat)
- All Milk—sum of whole, reduced-fat, low-fat and non-fat milks

Non-consumers were defined as subjects not consuming any specific type of milk during the 24-h recall. Consumers of a specific type of milk were defined as subjects consuming that type of milk and no other milk during the 24-h recall. Subjects consuming more than one type of milk during the 24-h recall were designated as “Mixed Milk-Consumers”. Consumer intake tertiles were calculated within each age/gender group (Table 1).

### 2.3. Serum Vitamin D

Serum 25(OH)D concentrations were obtained from NHANES laboratory files [22]. Briefly, NHANES measured serum 25(OH)D, using a standardized liquid chromatography–tandem mass (LC-MS/MS) method for 2007–2010 cycles and using a DiaSorin RIA kit for 2001–2006 cycles. RIA measurements of 25(OH)D concentration were later converted to LC-MS/MS method equivalent measurements adjusting for assay drifts, due to concerns about imprecision and bias in the method [23].

### 2.4. Statistics

All analyses were performed using SAS 9.2, 9.4 and SUDAAN 11. Day 1 weights were used all analyses and the data were adjusted for the complex sampling design of NHANES, using appropriate survey weights, strata, and primary sampling units. Separate analyses were conducted for the ages 2–8, 9–18, 19–70, and 71+ years. Least Square Means (LSM) were generated from models for each age/gender/ethnic group using linear regression across tertiles of milk intake and different types of milk intake after adjusting for age, gender, ethnicity, poverty income ratio and BMI or BMI Z-score when the population being analyzed was <19 years. Significance was set at *p* < 0.05. Logistic regression analysis was used to assess odds ratios (OR) and 95% confidence limits (Lower confidence limit (LCL); Upper confidence limit (UCL)) of meeting recommended levels of serum vitamin D (>50 nmol/L) associated with milk intake with non-consumers as reference group after adjusting for age, gender, ethnicity, poverty income ratio and BMI or BMI Z-score when the population being analyzed was <19 years old. Additionally, vitamin D from dietary supplements, seafood, and other non-milk vitamin D sources were also subsequently added to models to assess whether the intake of these variables impacted the association of milk with serum vitamin D (serum 25(OH)D) levels.

## 3. Results

Approximately 80% children aged 2–8 years, 57% children aged 9–18 years, 42% adults aged 19–70 years and 60% adults aged 71+ years were milk consumers. All milk (sum of all milk types) consumption was higher in child consumers (2–18 years), compared to adult consumers (19+ years). Adult consumers aged 71+ years consumed about 37% less milk than children consumers aged 9–18 years (1.12 cup eq/d vs. 1.77 cup eq/d, respectively) (Table 2). Consumption of whole milk compared to non-fat milk was about 21% greater among children consumers aged 2–8 years but was 19% less among adult consumers aged 71+ years. Consumption of whole milk was similar to that of non-fat milk for children consumers aged 9–18 years and for adult consumers aged 19–70 years (Table 2). Depending upon ethnicity, milk contributed about 61–71% among those aged 2–8 years, 49–62% among those aged 9–18 years, 24–42% among those aged 19–70 years, and 28–48% among those aged 71+ years of total vitamin D intake (Table 3).

Serum vitamin D (serum 25(OH)D) was positively associated with milk intake in children aged 9–18 years and adults aged 19+ years (*p*_linear trend_ < 0.05). The association was also significant for both males and females of age 9+ years (*p*_linear trend_ < 0.05), except for 9–18-year-old males and for 71+ year- old females (*p*_linear trend_ > 0.05) (Table 4). In children aged 2–8 years, consumers of whole milk, reduced fat milk, and low-fat milk had significantly higher (*p* < 0.05) serum vitamin D (serum 25(OH)D) levels than non-consumers. Similarly, in children aged 9–18 years and adults aged 19+ years, consumers of reduced-fat milk, low-fat milk, and non-fat milk had higher (*p* < 0.05) serum vitamin D (serum 25(OH)D) levels than non-consumers of same age group. Children (aged 9–18 years) and adult (aged 19–70 years) consumers of mixed milk also had higher (*p* < 0.05) serum vitamin D (serum 25(OH)D) than respective non-consumers (Table 5). Adjusting for data in Table 2 and Table 3 for dietary supplements, seafood, and other non-milk vitamin D sources did not change these results (Table 4 and Table 5).

Consumption of all milk (for all ages—i.e., 2+ years), whole milk (for ages 19–70 years), reduced-fat milk (for ages 9–70 years), low-fat milk (for those aged 19+ years), and non-fat milk (for those aged 9+ years) was associated with significantly higher probability of meeting serum vitamin D recommendations when the analysis was conducted by milk amount (Table 6). Consumers of whole milk (of aged 2–8 years), reduced-fat milk (of all ages), low-fat milk (of all ages), non-fat milk (of aged 19+ years) and mixed milk (of aged 2–70 years) had a higher probability of meeting serum vitamin D recommendations in the analysis by milk type (Table 6).

Table 7 shows the serum vitamin D (serum 25(OH)D) status by tertiles of all milk intake among different ethnic populations. Serum vitamin D (serum 25(OH)D) was positively associated with milk intake among children aged 9–18 years and adults aged 19+ years of Mexican American and of “other” ethnicity (*p*_linear trend_ < 0.05). The increase in serum vitamin D (serum 25(OH)D) status with increasing milk intake was also significant (*p*_linear trend_ < 0.05) among non-Hispanic Whites aged 19+ years, and non-Hispanic Blacks aged 2–18 years. Additional adjustment for dietary supplements, seafood, and other non-milk vitamin D sources did not change the results (Table 7).

Table 8 presents the data on vitamin D status by milk type across different ethnic groups. Significantly (*p* < 0.05) higher levels of serum vitamin D (serum 25(OH)D) levels were observed for consumers of whole milk, reduced fat milk, low-fat milk, non-fat milk and mixed milk among the different age and ethnic groups examined (Table 6). Adjusting the data for dietary supplements, seafood, and other non-milk vitamin D sources did not modify the results (Table 8).

## 4. Discussion

The current cross-sectional analysis of data from the NHANES 2001–2010 demonstrated a significant association between milk consumption and serum vitamin D (serum 25(OH)D) status. Additionally, the results showed that the probability of meeting the vitamin D recommendations was greater in milk consumers vs. non-consumers. To the best of our knowledge, this is first analysis of nationally representative, non-institutionalized population of US children and adults examining the association of milk intake with vitamin D levels.

Poor vitamin D status (low serum 25(OH)D levels) is a global public health concern as over 50% of population has less than adequate serum vitamin D status [24]. In the US, about 18% of the US population aged 1+ years had insufficient serum vitamin D levels and were at risk of inadequacy (less than 50 nmol/L) according a recent analysis of NHANES 2011–2014 [15]. Liu et al. [25] estimated the prevalence of inadequate serum vitamin D levels in US adults to be 28.9% for VDD and 41.4% for VDI, from analysis of NHANES 2001–2010 and using the criteria recommended by the Endocrinology Society to define VDD as 25(OH)D <50 nmol/L and VDI as 25(OH)D <75 nmol/L [26]. The present analysis showed that the average serum vitamin D levels ranged from 64 to 75 nmol/L depending on age and gender in representative population of US children and adults aged 2+ years. These average serum vitamin D levels are well with in the 50–75 nmol/L range and are considered sufficient by IOM definition [1]. Vitamin D is a “nutrient of public health concern” as it is under-consumed to an extent that may lead to adverse health outcomes and higher intake of food sources are encouraged by DGA [17].

In the present analysis, serum Vitamin D levels were significantly associated with the intake of milk, depending on the type. Milk consumers, especially those of low fat and reduced fat milk, had higher probability of meeting >50 nmol/L serum vitamin D level benchmark set by IOM [1] than non-consumers; however, the mean serum vitamin D levels were always higher than 50 nmol/L. Although milk contains a low amount of naturally occurring vitamin D, almost all milk in the US is fortified with 100 IU/cup vitamin D irrespective of the type of milk [1,19,21]. Effectiveness of milk and other fortified foods in improving serum vitamin D status has been demonstrated in both clinical and observational studies (see [27,28] for reviews). A recent systematic review and meta-analysis of randomized controlled trials showed that vitamin D fortified foods (mostly milk and dairy products) increased serum vitamin D levels by 1.2 nmol/L for each 1 µg/d increased intake of vitamin D [27]. A cup of milk/d provides ∼2.5 µg of vitamin D. A review of observational studies also concluded that the intake of vitamin D fortified milk products was positively associated with vitamin D intake and serum vitamin D status and the association was stronger in countries with a national vitamin D fortification policy [28]. However, this review included only five studies from US which had small sample sizes and included only certain population groups. In our present analysis, the intake of certain milk types (especially of whole milk) was not associated with an increase in serum vitamin D levels in all population sub-groups, which is not immediately understood.

Age and ethnicity have been shown to affect serum vitamin D status [14,15,29,30]. Vitamin D serum levels generally decrease with age, and non-Hispanic Blacks have the lowest vitamin D levels or highest prevalence of VDD, followed by Hispanics and Asians [14,15,29,30]. In contrast, non-Hispanic Whites have the highest vitamin D levels or lowest prevalence of VDD [14,15,29,30]. Lower intake of milk with age, which was also observed in our study, could potentially explain the inverse association of age with vitamin D. In regard to ethnicity and vitamin D status, differences in milk and overall vitamin D intake as well as skin pigmentation and other factors are potentially responsible for the ethnic differences in vitamin D status [31]. For instance, studies have shown lower milk intake among non-Hispanic Blacks compared to non-Hispanic Whites [32]. In the present analysis, serum vitamin D status was associated with both type and amount of milk intake depending upon the age and ethnicity. However, the effect of gender on serum vitamin D status has been reported to be insignificant or inconsistent [14,15,30], but the associations between amount of milk intake and serum vitamin D status were mostly significant for both males and females in the present analysis. Therefore, continuing to encourage an increase in milk intake, especially among populations with VDD or VDI, could be an effective strategy to improve vitamin D status.

In addition to providing vitamin D, milk and dairy products, make significant nutrient contributions including nutrients under-consumed by most Americans—calcium and potassium—as well as magnesium, phosphorus, zinc, vitamin A, vitamin B12, riboflavin (B2), choline, high-quality protein and saturated fat; as such, the inclusion of dairy foods into healthy dietary patterns is associated with improving diet quality and reducing risk of obesity and chronic diseases [17,18,33,34,35,36].

A major limitation of our study is the inability to determine a cause–effect relationship due to the cross-sectional design of NHANES. Additionally, as with any study based on self-reported data, under- or over-reporting cannot be ruled out. Additionally, the results from this study may not specifically reflect the effect of milk consumption on vitamin D status, although we used vitamin D from dietary supplements, seafood, and other non-milk sources as covariates to adjust some of our results. Strengths of this study included the use of a large nationally representative sample achieved through combining several sets of NHANES data releases and adjusting for numerous covariates, but even with these covariates, some residual confounding may still exist.

## 5. Conclusions

In conclusion, the results of this study indicate that milk consumers consistently have higher serum vitamin D levels and higher probability of meeting the recommended levels. Vitamin D has been identified as a “nutrient of public health concern in the US” and, therefore, increasing the intake of milk (especially low-fat and reduced-fat) should be encouraged. Other sources of vitamin D may also help in improving vitamin D status.

## Figures and Tables

**Table 1:** Milk intake tertiles (cup eq/day) by milk type in different age groups, NHANES 2001–2010.

Milk Type	Age 2–8 Years	Age 9–18 Years	Age 19–70 Years	Age 71+ Years
Total Milk				
Tertile 1	<1.01	<1.02	<0.75	<0.50
Tertile 2	1.01 to <2.00	1.02 to <2.00	0.75 to <1.50	0.50 to <1.20
Tertile 3	≥2.00	≥2.00	≥1.50	≥1.20
Whole Milk				
Tertile 1	<0.92	<0.99	<0.50	<0.47
Tertile 2	0.92 to <1.62	0.99 to <1.62	0.50 to <1.41	0.47 to <1.06
Tertile 3	≥1.62	≥1.62	≥1.41	≥1.06
Reduced-Fat Milk				
Tertile 1	<0.96	<1.00	<0.75	<0.50
Tertile 2	0.96 to <1.82	1.00 to <1.78	0.75 to <1.44	0.50 to <1.04
Tertile 3	≥1.82	≥1.78	≥1.44	≥1.04
Low-Fat Milk				
Tertile 1	<0.99	<0.98	<0.74	<0.61
Tertile 2	0.99 to <1.39	0.98 to <1.87	0.74 to <1.50	0.61 to <1.29
Tertile 3	≥1.39	≥1.87	≥1.50	≥1.29
Non-Fat Milk				
Tertile 1	<0.76	<1.00	<0.63	<0.50
Tertile 2	0.76 to <1.45	1.00 to <1.43	0.63 to <1.43	0.50 to <1.13
Tertile 3	≥1.45	≥1.43	≥1.43	≥1.13

Mean usual intake ± standard error (SE). Intake of milk was assessed using the sum of Food Patterns Equivalents Database (FPED) variable “D_Milk” as cup equivalents/day from associated What We Eat In America (WWEIA) categories: Whole Milk—1002 (Milk, Whole), 1202 (Flavored milk, whole); Reduced-fat Milk—1004 (Milk, reduced-fat), 1204 (Flavored milk, reduced-fat); Low-fat Milk—1006 (Milk, low-fat), 1206 (Flavored milk, low-fat); Non-fat Milk—1008 (Milk, nonfat), 1208 (Flavored milk, non-fat); All Milk—sum of whole, reduced-fat, low-fat and non-fat milks.

**Table 2:** Mean intake of milk (cup eq/day) by milk type in different age and gender groups, NHANES 2001–2010.

Milk Type	Age 2–8 Years		Age 9–18 Years		Age 19–70 Years		Age 71+ Years	
	Total Population	Consumer	Total Population	Consumer	Total Population	Consumer	Total Population	Consumer
All Milk								
All	1.40 ± 0.03	1.71 ± 0.03	1.11 ± 0.03	1.77 ± 0.04	0.62 ± 0.02	1.41 ± 0.03	0.69 ± 0.02	1.12 ± 0.03
Male	1.51 ± 0.04	1.82 ± 0.04	1.33 ± 0.05	2.00 ± 0.06	0.72 ± 0.02	1.63 ± 0.04	0.78 ± 0.08	1.24 ± 0.05
Female	1.2 8 ± 0.04	1.58 ± 0.04	0.89 ± 0.03	1.50 ± 0.04	0.52 ± 0.02	1.19 ± 0.03	0.63 ± 0.02	1.03 ± 0.03
Whole Milk								
All	0.46 ± 0.03	1.50 ± 0.04	0.27 ± 0.02	1.58 ± 0.06	0.15 ± 0.01	1.38 ± 0.05	0.12 ± 0.01	0.94 ± 0.05
Male	0.49 ± 0.03	1.59 ± 0.06	0.34 ± 0.02	1.75 ± 0.08	0.19 ± 0.01	1.60 ± 0.08	0.16 ± 0.02	1.18 ± 0.07
Female	0.43 ± 0.03	1.40 ± 0.05	0.20 ± 0.01	1.34 ± 0.06	0.12 ± 0.01	1.12 ± 0.04	0.09 ± 0.01	0.73 ± 0.05
Reduced-Fat Milk								
All	0.61 ± 0.04	1.55 ± 0.05	0.47 ± 0.02	1.62 ± 0.05	0.23 ± 0.01	1.36 ± 0.04	0.27 ± 0.01	1.04 ± 0.04
Male	0.67 ± 0.05	1.65 ± 0.06	0.58 ± 0.04	1.87 ± 0.08	0.28 ± 0.01	1.55 ± 0.06	0.31 ± 0.02	1.15 ± 0.06
Female	0.53 ± 0.03	1.43 ± 0.05	0.36 ± 0.02	1.32 ± 0.05	0.17 ± 0.01	1.13 ± 0.04	0.24 ± 0.02	0.94 ± 0.04
Low-Fat Milk								
All	0.17 ± 0.02	1.36 ± 0.06	0.16 ± 0.01	1.65 ± 0.07	0.09 ± 0.01	1.37 ± 0.06	0.12 ± 0.01	1.14 ± 0.05
Male	0.17 ± 0.02	1.42 ± 0.09	0.17 ± 0.02	1.69 ± 0.09	0.09 ± 0.01	1.60 ± 0.11	0.12 ± 0.01	1.15 ± 0.06
Female	0.17 ± 0.02	1.29 ± 0.06	0.15 ± 0.02	1.60 ± 0.10	0.08 ± 0.01	1.18 ± 0.05	0.12 ± 0.01	1.09 ± 0.06
Non-Fat Milk								
All	0.16 ± 0.02	1.24 ± 0.08	0.20 ± 0.02	1.55 ± 0.09	0.15 ± 0.01	1.37 ± 0.05	0.19 ± 0.01	1.16 ± 0.05
Male	0.18 ± 0.03	1.26 ± 0.10	0.23 ± 0.03	1.78 ± 0.14	0.16 ± 0.01	1.60 ± 0.08	0.19 ± 0.02	1.26 ± 0.07
Female	0.15 ± 0.03	1.21 ± 0.04	0.17 ± 0.02	1.31 ± 0.08	0.14 ± 0.01	1.18 ± 0.05	0.19 ± 0.01	1.09 ± 0.06

Mean usual intake ± standard error (SE). Intake of milk was assessed using the sum of Food Patterns Equivalents Database (FPED) variable “D_Milk” as cup equivalents/day from associated What We Eat In America (WWEIA) categories: Whole Milk—1002 (Milk, Whole), 1202 (Flavored milk, whole); Reduced-fat Milk—1004 (Milk, reduced-fat), 1204 (Flavored milk, reduced-fat); Low-fat Milk—1006 (Milk, low-fat), 1206 (Flavored milk, low-fat); Non-fat Milk—1008 (Milk, nonfat), 1208 (Flavored milk, non-fat); All Milk—sum of whole, reduced-fat, low-fat and non-fat milks. *N* = 4061 aged 2–8 years; 8700 aged 9–18 years; 17,457 aged 19–70 years; 3454 aged 71–99 years.

**Table 3:** Estimated mean dietary intake of vitamin D (µg/day) by milk type and by age and ethnicity. NHANES 2001–2010 Day 1 dietary data.

	Mexican American	Other Hispanic	Non-Hispanic White	Non-Hispanic Black	Other
2–8 years					
All Milk	4.81 ± 0.13	5.47 ± 0.31	4.47 ± 0.12	3.29 ± 0.12	4.22 ± 0.17
Whole Milk	2.43 ± 0.15	2.83 ± 0.27	1.28 ± 0.10	1.72 ± 0.11	1.82 ± 0.21
Reduced-Fat	1.71 ± 0.10	1.75 ± 0.25	1.96 ± 0.11	1.17 ± 0.09	1.90 ± 0.22
Low-Fat Milk	0.36 ± 0.05	0.70 ± 0.18	0.62 ± 0.06	0.23 ± 0.05	0.33 ± 0.09
Non-Fat Milk	0.31 ± 0.05	0.20 ± 0.05	0.61 ± 0.08	0.17 ± 0.03	0.17 ± 0.06
Not Milk	2.16 ± 0.06	2.43 ± 0.17	1.87 ± 0.06	2.14 ± 0.07	2.08 ± 0.12
9–18 years					
All Milk	3.03 ± 0.13	2.90 ± 0.21	3.71 ± 0.14	2.13 ± 0.07	2.52 ± 0.23
Whole Milk	1.00 ± 0.07	1.38 ± 0.20	0.75 ± 0.07	0.98 ± 0.07	0.71 ± 0.12
Reduced-Fat	1.27 ± 0.07	0.87 ± 0.10	1.61 ± 0.10	0.75 ± 0.05	1.21 ± 0.18
Low-Fat Milk	0.36 ± 0.04	0.27 ± 0.05	0.62 ± 0.06	0.18 ± 0.03	0.21 ± 0.05
Non-Fat Milk	0.39 ± 0.07	0.38 ± 0.11	0.73 ± 0.07	0.22 ± 0.03	0.39 ± 0.08
Not Milk	2.25 ± 0.06	2.36 ± 0.16	2.32 ± 0.06	2.26 ± 0.07	2.40 ± 0.20
19–70 years					
All Milk	1.83 ± 0.08	1.49 ± 0.08	2.05 ± 0.06	0.92 ± 0.05	1.46 ± 0.17
Whole Milk	0.82 ± 0.06	0.59 ± 0.07	0.43 ± 0.03	0.46 ± 0.04	0.49 ± 0.08
Reduced-Fat	0.75 ± 0.06	0.58 ± 0.06	0.74 ± 0.03	0.36 ± 0.03	0.70 ± 0.15
Low-Fat Milk	0.12 ± 0.02	0.15 ± 0.03	0.32 ± 0.03	0.06 ± 0.01	0.15 ± 0.06
Non-Fat Milk	0.14 ± 0.02	0.17 ± 0.05	0.55 ± 0.04	0.04 ± 0.01	0.12 ± 0.02
Not Milk	2.63 ± 0.09	2.60 ± 0.16	2.85 ± 0.06	2.84 ± 0.11	2.79 ± 0.19
71+ years					
All Milk	1.99 ± 0.14	2.04 ± 0.27	2.17 ± 0.07	1.16 ± 0.12	1.46 ± 0.31
Whole Milk	0.72 ± 0.09	1.01 ± 0.23	0.34 ± 0.03	0.45 ± 0.09	0.27 ± 0.11
Reduced-Fat	0.90 ± 0.12	0.53 ± 0.15	0.83 ± 0.05	0.50 ± 0.06	0.65 ± 0.15
Low-Fat Milk	0.12 ± 0.04	0.06 ± 0.04	0.41 ± 0.04	0.12 ± 0.03	0.09 ± 0.05
Non-Fat Milk	0.26 ± 0.05	0.44 ± 0.15	0.59 ± 0.04	0.10 ± 0.02	0.46 ± 0.27

Mean usual intake ± standard error (SE). Milk type was assessed using the sum of Food Patterns Equivalents Database (FPED) variable “D_Milk” as cup equivalents/day for What We Eat In America (WWEIA) categories: Whole Milk—1002 (Milk, Whole), 1202 (Flavored milk, whole); Reduced-fat Milk—1004 (Milk, reduced-fat), 1204 (Flavored milk, reduced-fat); Low-fat Milk—1006 (Milk, low-fat), 1206 (Flavored milk, low-fat); Non-fat Milk—1008 (Milk, nonfat), 1208 (Flavored milk, non-fat); All Milk—sum of whole, reduced-fat, low-fat and non-fat milks. *N* = 42,154.

**Table 4:** Serum Vitamin D (serum 25(OH)D) status (nmol/L) by all milk intake in different age and gender groups, NHANES 2001–2010.

	All	Non-Consumers	Consumer Tertile 1	Consumer Tertile 2	Consumer Tertile 3	*p*_linear trend_	*p*_linear trend_^a^
2–8 years							
All	74.5 ± 0.7	71.7 ± 1.6	74.5 ± 1.1	75.4 ± 1.0 *^#^	75.4 ± 0.8 *^#^	0.1721	0.1541
Male	75.0 ± 0.8	71.8 ± 2.0	74.9 ± 1.1	76.9 ± 1.3 *^#^	75.7 ± 1.0	0.6085	0.5164
Female	73.9 ± 0.9	71.7 ± 2.0	73.2 ± 1.6	74.9 ± 1.2	74.8 ± 1.0	0.0609	0.0624
9–18 years							
All	65.7 ± 0.8	63.5 ± 1.1	65.7 ± 1.0 *	66.1 ± 1.0 *^#^	69.3 ± 0.9 *^#^	0.0034	0.0049
Male	67.3 ± 0.8	64.1 ± 1.3	67.7 ± 1.3 *^#^	68.2 ± 1.2 *^#^	71.1 ± 1.1 *^#^	0.0505	0.0478
Female	63.9 ± 0.8	62.6 ± 1.2	64.2 ± 1.1	64.6 ± 1.4	66.3 ± 1.2 *	0.0123	0.0267
19–70 years							
All	64.4 ± 0.5	62.9 ± 0.6	64.3 ± 0.8	66.8 ± 0.7 *^#^	67.8 ± 0.6 *^#^	<0.0001	<0.0001
Male	63.9 ± 0.5	62.5 ± 0.6	64.0 ± 0.9	65.8 ± 0.8 *^#^	67.2 ± 0.6 *^#^	0.0004	0.0002
Female	64.8 ± 0.6	63.1 ± 0.7	65.4 ± 1.0 *^#^	67.5 ± 1.1 *^#^	68.5 ± 0.9 *^#^	0.0109	0.0128
71+ years							
All	65.8 ± 0.9	63.0 ± 1.2	67.2 ± 1.6 *^#^	65.4 ± 1.1	70.2 ± 1.1 *^#^	0.0039	0.0001
Male	65.7 ± 0.9	64.3 ± 1.3	65.2 ± 1.7	64.5 ± 1.3	70.1 ± 1.4 *^#^	0.0049	0.0010
Female	65.9 ± 1.0	62.0 ± 1.4	68.4 ± 1.8 *^#^	66.3 ± 1.5 *	70.1 ± 1.5 *^#^	0.1658	0.0229

Mean serum vitamin D concentration ± standard error (SE). Least Square Means (LSM) were modeled for each age/gender group using linear regression across tertiles after adjusting the data for age, gender, ethnicity, poverty income ratio and BMI or BMI Z-score when the population being analyzed was < 19 years. * Significant difference from non-consumer at *p* < 0.05. ^#^ Significant difference from non-consumer at *p* < 0.05 after additionally adjusting data for vitamin D intake from dietary supplements, sea food, and other non-milk dietary sources. ^a^ *p*_linear trend_ after additionally adjusting data for vitamin D intake from non-milk sources, supplements and sea food. *N* = 4061 aged 2–8 years; 8700 aged 9–18 years; 17,457 aged 19–70 years; 3454 aged 71–99 years.

**Table 5:** Serum vitamin D (serum 25(OH)D) status (nmol/L) by different types of milk intake in different age groups, NHANES 2001–2010.

Type of Milk	Age 2–8 Years	Age 9–18 Years	Age 19–70 Years	Age 71+ Years
Total Population	74.5 ± 0.7	65.7 ± 0.8	64.4 ± 0.5	65.8 ± 0.9
Non-Consumer	71.7 ± 1.6	63.6 ± 1.1	62.9 ± 0.6	63.0 ± 1.2
Whole Milk Consumer	75.4 ± 1.2 *^#^	65.3 ± 1.2	63.7 ± 0.8	65.7 ± 2.0
Reduced-Fat Milk Consumer	74.9 ± 0.9 *^#^	66.5 ± 0.9 *^#^	66.4 ± 0.6 *^#^	66.8 ± 1.3 *^#^
Low-Fat Milk Consumer	78.2 ± 2.2 *^#^	68.0 ± 1.4 *^#^	67.8 ± 1.1 *^#^	70.2 ± 1.5 *^#^
Non-Fat Milk Consumer	73.4 ± 1.9	68.1 ± 1.6 *^#^	68.3 ± 0.8 *^#^	68.7 ± 1.2 *^#^
Mixed Milk Consumer	73.2 ± 1.3	69.3 ± 1.0 *^#^	67.6 ± 1.9 *^#^	65.5 ± 2.3

Mean serum vitamin D concentration ± standard error (SE). Least Square Means (LSM) were modeled for each age group using linear regression across different types of milk intake after adjusting gender combined data for age, gender, ethnicity, poverty income ratio and BMI or BMI Z-score when the population being analyzed was < 19 years. * Significant difference from non-consumer at *p* < 0.05. ^#^ Significant difference from non-consumer at *p* < 0.05 after additionally adjusting data for vitamin D intake from dietary supplements, sea food, and other non-milk dietary sources. *N* = 4061 aged 2–8 years; 8700 aged 9–18 years; 17,457 aged 19–70 years; 3454 aged 71–99 years.

**Table 6:** Odds ratios (OR), 95% confidence limits (Lower confidence limit (LCL)/Upper confidence limit (UCL)) for meeting the recommended serum vitamin D (>50 nmol/L), NHANES 2001–2010.

	OR (LCL, UCL) by Milk Amount	OR (LCL, UCL) by Milk Type
2–8 years		
All Milk	1.42 (1.19, 1.69)	--
Whole Milk Consumer	1.21 (0.98, 1.50)	2.06 (1.46, 2.89)
Reduced-Fat Milk Consumer	1.24 (0.97, 1.57)	1.88 (1.21, 2.91)
Low-Fat Milk Consumer	1.40 (0.80, 2.45)	3.15 (1.34, 7.39)
Non-Fat Milk Consumer	1.29 (0.81, 2.05)	1.67 (0.88, 3.17)
Mixed Milk	--	3.77 (2.06, 6.90)
9–18 years		
All Milk	1.31 (1.20, 1.44)	----
Whole Milk Consumer	1.07 (0.95, 1.21)	1.22 (0.96, 1.54)
Reduced-Fat Milk Consumer	1.34 (1.19, 1.50)	1.77 (1.38, 2.27)
Low-Fat Milk Consumer	1.20 (0.90, 1.59)	1.48 (1.02, 2.14)
Non-Fat Milk Consumer	1.36 (1.08, 1.70)	1.49 (0.90, 2.47)
Mixed Milk	----	2.66 (1.91, 3.71)
19–70 years		
All Milk	1.31 (1.23, 1.41)	----
Whole Milk Consumer	1.12 (1.04, 1.20)	1.20 (1.00, 1.43)
Reduced-Fat Milk Consumer	1.27 (1.16, 1.39)	1.59 (1.39, 1.82)
Low-Fat Milk Consumer	1.58 (1.24, 2.03)	2.22 (1.61, 3.06)
Non-Fat Milk Consumer	1.31 (1.06, 1.63)	1.90 (1.53, 2.36)
Mixed Milk	----	2.10 (1.43, 3.09)
71+ years		
All Milk	1.35 (1.17, 1.56)	-
Whole Milk Consumer	1.08 (0.92, 1.27)	1.13 (0.87, 1.47)
Reduced-Fat Milk Consumer	1.18 (0.96, 1.44)	1.46 (1.10, 1.93)
Low-Fat Milk Consumer	1.67 (1.24, 2.24)	2.33 (1.59, 3.43)
Non-Fat Milk Consumer	1.29 (1.05, 1.59	1.77 (1.31, 2.39)
Mixed Milk	-	1.38 (0.73, 2.59)

Gender combined data. OR were estimated using logistic regressions to model meeting recommended serum vitamin d (>50 nmol/L) on milk intake (OR Amount) or on 6 types of milk consumers (OR Type). Non-consumers were the reference group in both estimations. *N* = 4061 aged 2–8 years; 8700 aged 9–18 years; 17,457 aged 19–70 years; 3454 aged 71–99 years.

**Table 7:** Serum vitamin D (serum 25(OH)D) status (nmol/L) by all milk intake in ethnic population groups, NHANES 2001–2010.

	All	Non-Consumers	Consumer Tertile 1	Consumer Tertile 2	Consumer Tertile 3	*p*_linear trend_	*p*_linear trend_^a^
Mexican American							
2–8 years	67.6 ± 0.7	63.5 ± 1.3	67.8 ± 1.3 *^#^	68.0 ± 1.0 *^#^	69.6 ± 1.1 *^#^	0.0929	0.1458
9–18 years	57.4 ± 0.8	54.1 ± 1.0	57.2 ± 1.1 *^#^	59.7 ± 1.1 *^#^	61.0 ± 1.1 *^#^	0.0004	0.0010
19–70 years	54.2 ± 0.8	53.4 ± 0.9	53.8 ± 1.3	53.9 ± 1.2	57.8 ± 1.3 *^#^	0.0024	0.0064
71+ years	56.0 ± 1.5	55.9 ± 2.3	50.4 ± 2.4 ^#^	56.6 ± 2.5	60.6 ± 2.2 ^#^	0.0018	<0.0001
Other Hispanics							
2–8 years	69.9 ± 1.3	64.8 ± 2.0	67.1 ± 2.1	73.9 ± 2.8 *^#^	72.2 ± 1.9 *^#^	0.0199	0.0348
9–18 years	60.5 ± 1.2	58.2 ± 1.5	59.5 ± 1.9	61.9 ± 2.2	64.2 ± 3.0	0.0755	0.0517
19–70 years	57.1 ± 1.2	55.9 ± 1.6	57.1 ± 1.2	60.2 ± 2.1	58.2 ± 1.6	0.5415	0.3929
71+ years	63.7 ± 2.2	57.8 ± 2.7	61.4 ± 3.9	66.0 ± 4.9	74.7 ± 6.9 *^#^	<0.0001	<0.0001
Non-Hispanic White							
2–8 years	80.4 ± 1.2	78.1 ± 2.6	81.8 ± 1.7	80.7 ± 1.7	80.3 ± 1.2	0.8450	0.9377
9–18 years	72.7 ± 1.1	71.2 ± 1.5	73.1 ± 1.5	72.6 ± 1.4	75.3 ± 1.2 *^#^	0.0645	0.0843
19–70 years	70.0 ± 0.6	68.4 ± 0.7	69.6 ± 1.0	72.6 ± 0.9 *^#^	73.4 ± 0.7 *^#^	0.0006	0.0006
71+ years	67.6 ± 0.9	64.7 ± 1.3	69.5 ± 1.7 *^#^	67.4 ± 1.2	71.5 ± 1.2 *^#^	0.0217	0.0006
Non-Hispanic Black							
2–8 years	61.4 ± 0.8	59.7 ± 1.6	57.4 ± 1.3	63.1 ± 1.4	64.6 ± 1.3 *^#^	<0.0001	<0.0001
9–18 years	48.3 ± 0.8	43.9 ± 1.2	49.0 ± 1.0 *^#^	48.7 ± 1.0 *^#^	55.0 ± 1.2 *^#^	0.0288	0.0332
19–70 years	44.2 ± 0.8	41.8 ± 0.9	46.8 ± 1.3 *^#^	47.7 ± 1.3 *^#^	47.2 ± 1.3 *^#^	0.6355	0.4175
71+ years	51.9 ± 1.8	48.7 ± 2.0	54.5 ± 3.1	50.6 ± 2.8	57.1 ± 4.9	0.6749	0.5408
Other							
2–8 years	70.1 ± 1.6	65.5 ± 3.1	68.7 ± 2.8	73.4 ± 1.8 *^#^	71.4 ± 2.1	0.1000	0.0419
9–18 years	56.5 ± 1.2	54.8 ± 2.1	52.2 ± 2.7	57.0 ± 2.5	63.0 ± 2.2 *^#^	0.0019	0.0011
19–70 years	52.8 ± 1.1	51.7 ± 1.4	50.6 ± 2.2	53.4 ± 2.8	58.5 ± 2.4 *^#^	0.0004	<0.0001
71+ years	56.9 ± 2.6	56.7 ± 3.6	51.8 ± 5.1	51.5 ± 6.4	67.6 ± 4.9	0.0067	0.0218

Mean serum vitamin D concentration ± standard error (SE). Least Square Means (LSM) were modeled for each age/ethnic group using linear regression across tertiles of milk intake after adjusting gender combined data for age, gender, poverty income ratio and BMI or BMI Z-score when the population being analyzed was < 19 years. * Significant difference from non-consumer at *p* < 0.05. ^#^ Significant difference from non-consumer at *p* < 0.05 after additionally adjusting data for vitamin D intake from non-milk sources, supplements and sea food. ^a^ *p*_linear trend_ after additionally adjusting data for vitamin D intake from non-milk sources, supplements and sea food. N = 7827 Mexican American; 2094 Other Hispanic; 14,525 non-Hispanic White; 7739 non-Hispanic Black; 1487 Other.

**Table 8:** Serum vitamin D (serum 25(OH)D) status (nmol/L) by milk type in ethnic population groups, NHANES 2001–2010.

	Mexican American	Other Hispanic	Non-Hispanic White	Non-Hispanic Black	Other
2–8 years					
Non-Consumer	63.4 ± 1.3	64.8 ± 2.0	78.2 ± 2.6	59.7 ± 1.6	65.5 ± 3.1
Whole Milk Consumer	69.2 ± 1.0 *^#^	71.1 ± 2.6 *^#^	81.6 ± 2.0	60.8 ± 1.4	69.7 ± 1.9
Reduced-Fat Milk Consumer	68.5 ± 1.1 *^#^	74.6 ± 2.9 *^#^	80.3 ± 1.4	60.7 ± 1.3	72.8 ± 3.3 *^#^
Low-Fat Milk Consumer	71.4 ± 1.8 *^#^	68.5 ± 3.5	84.7 ± 3.8	65.1 ± 3.4	72.2 ± 5.7
Non-Fat Milk Consumer	61.4 ± 2.9	70.3 ± 6.0	82.5 ± 2.9	53.0 ± 2.9	67.0 ± 1.9
Mixed Milk Consumer	67.3 ± 1.1 *^#^	66.3 ± 1.8	78.0 ± 2.1	64.2 ± 1.7 *^#^	69.2 ± 2.7
9–18 years					
Non-Consumer	54.0 ± 1.0	58.2 ± 1.5	71.2 ± 1.5	43.9 ± 1.2	54.8 ± 2.1
Whole Milk Consumer	57.5 ± 1.2 *^#^	64.1 ± 2.3 *^#^	71.6 ± 1.8	49.0 ± 1.0 *^#^	55.3 ± 2.4
Reduced-Fat Milk Consumer	59.3 ± 1.0 *^#^	58.9 ± 1.5	72.5 ± 1.2	52.0 ± 1.0 *^#^	60.3 ± 2.4
Low-Fat Milk Consumer	59.5 ± 1.6 *^#^	58.9 ± 2.0	76.3 ± 2.0 *^#^	49.0 ± 2.4	52.4 ± 6.3
Non-Fat Milk Consumer	62.9 ± 1.7 *^#^	60.9 ± 4.0	75.8 ± 2.3	47.7 ± 1.7 *^#^	53.3 ± 6.6
Mixed Milk Consumer	59.7 ± 1.7 *^#^	64.3 ± 3.7	75.9 ± 1.6 *^#^	56.1 ± 1.8 *^#^	56.7 ± 4.0
19–70 years					
Non-Consumer	53.3 ± 0.9	55.8 ± 1.6	68.4 ± 0.7	41.8 ± 0.9	51.7 ± 1.4
Whole Milk Consumer	53.5 ± 1.3	58.0 ± 1.7	68.8 ± 1.0	45.0 ± 0.9 *^#^	52.8 ± 2.6
Reduced-Fat Milk Consumer	56.6 ± 1.2 *^#^	57.1 ± 1.6	71.9 ± 0.8 *^#^	47.8 ± 1.5 *^#^	53.9 ± 2.3
Low-Fat Milk Consumer	56.1 ± 1.8	54.8 ± 2.9	73.0 ± 1.1 *^#^	52.9 ± 2.8 *^#^	56.4 ± 5.8
Non-Fat Milk Consumer	55.3 ± 2.4	65.0 ± 3.4 *^#^	73.2 ± 0.9 *^#^	51.2 ± 3.0 *^#^	60.2 ± 4.2
Mixed Milk Consumer	56.5 ± 2.6	61.0 ± 3.2	74.3 ± 2.5 *^#^	49.7 ± 3.5 *^#^	35.7 ± 4.4 *^#^
71+ years					
Non-Consumer	55.8 ± 2.3	57.6 ± 2.8	64.7 ± 1.3	48.6 ± 2.0	56.7 ± 3.6
Whole Milk Consumer	53.7 ± 1.8	65.8 ± 5.9	67.7 ± 2.3	49.3 ± 3.0	55.9 ± 6.4
Reduced-Fat Milk Consumer	57.9 ± 2.0	63.8 ± 3.7	68.3 ± 1.4 *^#^	58.3 ± 3.0 *^#^	53.0 ± 5.5
Low-Fat Milk Consumer	53.2 ± 9.0	70.1 ± 8.2	72.3 ± 1.5 *^#^	53.8 ± 4.4	67.1 ± 8.4
Non-Fat Milk Consumer	57.6 ± 4.9	77.9 ± 7.3 *^#^	71.1 ± 1.3 *^#^	43.6 ± 3.5	63.5 ± 6.8
Mixed Milk Consumer	55.1 ± 10.8	70.2 ± 6.8	65.6 ± 2.6	71.3 ± 6.2 *^#^	59.4 ± 4.4

Mean serum vitamin D concentration ± standard error (SE). Least Square Means (LSM) were modeled for each age/ethnic group using linear regression across different types of milk intake after adjusting gender combined data for age, gender, poverty income ratio and BMI or BMI Z-score when the population being analyzed was < 19 years. * Significant difference from non-consumer at *p* < 0.05. ^#^ Significant difference from non-consumer at *p* < 0.05 after additionally adjusting data for vitamin D intake from non-milk sources. *N* = 7827 Mexican American; 2094 Other Hispanic; 14,525 non-Hispanic White; 7739 non-Hispanic Black; 1487 Other.

## References

[1] Institute of Medicine, Food and Nutrition Board (2011). Dietary Reference Intakes for Calcium and Vitamin D.

[2] Holick M.F. (2007). Vitamin D deficiency. N. Engl. J. Med..

[3] Holick M.F. (2006). Resurrection of vitamin D deficiency and rickets. J. Clin. Investig..

[4] Tang B.M., Eslick G.D., Nowson C., Smith C., Bensoussan A. (2007). Use of calcium or calcium in combination with vitamin D supplementation to prevent fractures and bone loss in people aged 50 years and older: A meta-analysis. Lancet.

[5] Bischoff-Ferrari H.A., Dawson-Hughes B., Staehelin H.B., Orav J.E., Stuck A.E., Theiler R., Wong J.B., Egli A., Kiel D.P., Henschkowski J. (2009). Fall prevention with supplemental and active forms of vitamin D: A meta-analysis of randomized controlled trials. BMJ.

[6] Giovannucci E., Liu Y., Hollis B.W., Rimm E.B. (2008). 25-hydroxyvitamin D and risk of myocardial infarction in men: A prospective study. Arch. Intern. Med..

[7] Vaidya A., Forman J.P. (2010). Vitamin D and Hypertension: Current Evidence and Future Directions. Hypertension.

[8] Ford E.S., Ajani U.A., McGuire L.C., Liu S. (2005). Concentrations of serum vitamin D and the metabolic syndrome among U.S. adults. Diabetes Care.

[9] Munger K.L., Zhang S.M., O’Reilly E., Hernan M.A., Olek M.J., Willett W.C., Ascherio A. (2004). Vitamin D intake and incidence of multiple sclerosis. Neurology.

[10] Yin L., Ordonez-Mena J.M., Chen T., Schottker B., Arndt V., Brenner H. (2013). Circulating 25-hydroxyvitamin D serum concentration and total cancer incidence and mortality: A systematic review and meta-analysis. Prev. Med..

[11] Merlino L.A., Curtis J., Mikuls T.R., Cerhan J.R., Criswell L.A., Saag K.G. (2004). Iowa Women’s Health Study. Vitamin D intake is inversely associated with rheumatoid arthritis: Results from the Iowa Women’s Health Study. Arthritis Rheum.

[12] Hirani V., Cumming R.G., Naganathan V., Blyth F., Le Couteur D.G., Hsu B., Handelsman D.J., Waite L.M., Seibel M.J. (2017). Longitudinal Associations Between Vitamin D Metabolites and Sarcopenia in Older Australian men: The Concord Health and Aging in Men Project. J. Gerontol. A Biol. Sci. Med. Sci..

[13] Wei M.Y., Giovannucci E.L. (2010). Vitamin D and multiple health outcomes in the Harvard cohorts. Mol. Nutr. Food Res..

[14] U.S. Centers for Disease Control and Prevention Second National Report on Biochemical Indicators of Diet and Nutrition in the U.S. Population 2012.

[15] Herrick K.A., Storandt R.J., Afful J., Pfeiffer C.M., Schleicher R.L., Gahche J.J., Potischman N. (2019). Vitamin D status in the United States, 2011–2014. Am. J. Clin. Nutr..

[16] USDA, Agricultural Research Service Usual Nutrient Intake from Food and Beverages, by Gender and Age, What We Eat in America, NHANES 2013–2016. https://www.ars.usda.gov/ARSUserFiles/80400530/pdf/usual/Usual_Intake_gender_WWEIA_2013_2016.pdf.

[17] U.S. Department of Health and Human Services, U.S. Department of Agriculture (2015). 2015–2020 Dietary Guidelines for Americans.

[18] Dietary Guidelines Advisory Committee Scientific Report of the 2020 Dietary Guidelines Advisory Committee: Advisory Report to the Secretary of Agriculture and the Secretary of Health and Human Services.

[19] Calvo M.S., Whiting S.J., Preedy R.V., Srirajaskanthan R., Patel V. (2013). Vitamin D Fortification in North America: Current Status and Future Considerations. The Handbook of Food Fortification from Concepts to Public Health Applications.

[20] Calvo M.S., Whiting S.J., Barton C.N. (2004). Vitamin D fortification in the United States and Canada: Current status and data needs. Am. J. Clin. Nutr..

[21] Yetley E.A. (2008). Assessing the vitamin D status of the US population. Am. J. Clin. Nutr..

[22] Centers for Disease Control and Prevention (CDC), National Center for Health Statistics National Health and Nutrition Examination Survey.

[23] Schleicher R.L., Sternberg M.R., Lacher D.A., Sempos C.T., Looker A.C., Durazo-Arvizu R.A., Yetley E.A., Chaudhary-Webb M., Maw K.L., Pfeiffer C.M. (2016). A Method-bridging Study for Serum 25-hydroxyvitamin D to Standardize Historical Radioimmunoassay Data to Liquid Chromatography-Tandem Mass Spectrometry. Natl. Health Stat. Rep..

[24] Van Schoor N., Lips P. (2017). Global Overview of Vitamin D Status. Endocrinol. Metab. Clin. N. Am..

[25] Liu X., Baylin A., Levy P.D. (2018). Vitamin D deficiency and insufficiency among US adults: Prevalence, predictors and clinical implications. Br. J. Nutr..

[26] Holick M.F., Binkley N.C., Bischoff-Ferrari H.A., Gordon C.M., Hanley D.A., Heaney R.P., Murad M.H., Weaver C.M. (2011). Evaluation, treatment, and prevention of vitamin D deficiency: An Endocrine Society Clinical Practice Guideline. J. Clin. Endocrin. Metab..

[27] Black L.J., Seamans K.M., Cashman K.D., Kiely M. (2012). An updated systematic review and meta-analysis of the efficacy of vitamin D food fortification. J. Nutr..

[28] Itkonen S.T., Erkkola M., Lamberg-Allardt C.J.E. (2018). Vitamin D Fortification of Fluid Milk Products and Their Contribution to Vitamin D Intake and Vitamin D Status in Observational Studies-A Review. Nutrients.

[29] Forrest K.Y., Stuhldreher W.L. (2011). Prevalence and correlates of vitamin D deficiency in US adults. Nutr. Res..

[30] Parva N.R., Tadepalli S., Singh P., Qian A., Joshi R., Kandala H., Nookala V.K., Cheriyath P. (2018). Prevalence of Vitamin D Deficiency and Associated Risk Factors in the US Population (2011–2012). Cureus.

[31] O’Neill C.M., Kazantzidis A., Kiely M., Cox L., Meadows S., Goldberg G., Prentice A., Kift R., Webb A.R., Cashman K.D. (2017). A predictive model of serum 25-hydroxyvitamin D in UK white as well as black and Asian minority ethnic population groups for application in food fortification strategy development towards vitamin D deficiency prevention. J. Steroid. Biochem. Mol. Biol..

[32] Sebastian R.S., Goldman J.D., Wilkinson Enns C., LaComb R.P. (2010). Fluid Milk Consumption in the United States: What We Eat in America, NHANES 2005–2006.

[33] O’Neil C.E., Nicklas T.A., Fulgoni V.L. (2018). Food sources of energy and nutrients of public health concern and nutrients to limit with a focus on milk and other dairy foods in children 2 to 18 years of age: National Health and Nutrition Examination Survey, 2011–2014. Nutrients.

[34] O’Neil C.E., Keast D.R., Fulgoni V.L., Nicklas T.A. (2012). Food sources of energy and nutrients among adults in the US: NHANES 2003–2006. Nutrients.

[35] Hess J.M., Cifelli C.J., Fulgoni V.L. (2020). Energy and nutrient intake of Americans according to meeting current dairy recommendations. Nutrients.

[36] Thorning T.K., Raben A., Tholstrup T., Soedamah-Muthu S.S., Givens I., Astrup A. (2016). Milk and dairy products: Good or bad for human health? An assessment of the totality of scientific evidence. Food Nutr. Res..

